# Using RIGHT (Reporting Items for Practice Guidelines in Healthcare) to evaluate the reporting quality of WHO guidelines

**DOI:** 10.1186/s12961-020-00578-w

**Published:** 2020-07-08

**Authors:** Xiaoqin Wang, Qi Zhou, Yaolong Chen, Nan Yang, Kevin Pottie, Yujie Xiao, Yajing Tong, Liang Yao, Qi Wang, Kehu Yang, Susan L. Norris

**Affiliations:** 1 Michael G. DeGroote Institute for Pain Research and Care, McMaster University, Hamilton, Canada; 2 Evidence-Based Medicine Center, School of Basic Medical Sciences, Lanzhou University, Lanzhou, China; 3 WHO Collaborating Center for Guideline Implementation and Knowledge Translation, Lanzhou, China; 4 Chinese GRADE Center, Lanzhou, China; 5 The First School of Clinical Medicine, Lanzhou University, Lanzhou, China; 6 Bruyère Research Institute, University of Ottawa, Ottawa, Canada; 7 The Second Hospital of Lanzhou University, Lanzhou, China; 8 School of Public Health of Lanzhou University, Lanzhou, China; 9 Department of Health Research Methods, Evidence and Impact, McMaster University, Hamilton, ON Canada; 10 Quality of Norms and Standards Department, Science Division, World Health Organization, Geneva, Switzerland

**Keywords:** WHO, Practice guideline, RIGHT (Reporting Items for Practice Guidelines in HealThcare), Reporting quality

## Abstract

**Background:**

Without adequate reporting of research, valuable time and resources are wasted. In the same vein, adequate reporting of practice guidelines to optimise patient care is equally important. Our study examines the quality of reporting of published WHO guidelines, over time, using the RIGHT (Reporting Items for Practice Guidelines in HealThcare) reporting checklist.

**Methods:**

We examined English-language guidelines approved by the WHO Guidelines Review Committee from inception of the committee in 2007 until 31 December 2017. Pairs of independent, trained reviewers assessed the reporting quality of these guidelines. Descriptive data were summarised with frequencies and percentages.

**Results:**

We included 182 eligible guidelines. Overall, 25 out of the 34 RIGHT items were reported in 75% or more of the WHO guidelines. The reporting rates improved over time. Further, 90% of the guidelines reported document type in the title. The identification of evidence, the rationale for recommendations and the review process were reported in more than 80% of guidelines. The certainty of the evidence using the Grading of Recommendations, Assessment, Development and Evaluation (GRADE) system was assessed in 81% of the guidelines assessed. While 82% of guidelines reported funding sources, only 25% mentioned the role of funders.

**Conclusions:**

WHO guidelines provide adequate reporting of many of the RIGHT items and reporting has improved over time. WHO guidelines compare favourably to guidelines produced by other organisations. However, reporting can be further improved in a number of areas.

## Introduction

Clinical practice guidelines are statements that include recommendations intended to optimise patient care [1]. A trustworthy guideline should not only comply with rigorous methodology standards [2] but it should also have transparent, clear and complete reporting [3]. Inadequately reported research wastes time and resources [4], and can seriously distort the available evidence, compromise its usefulness and reliability, and may also mislead end users [5]. Reporting of key elements of practice guidelines helps users assess the trustworthiness of a guideline and is an important facilitator for uptake and implementation of recommendations. Frequently, however, the reporting quality of practice guidelines is unsatisfactory; for example, in 2000, Grilli et al. [6] found that only 33% of 431 guidelines reported the type of stakeholders involved in guideline development, 18% reported the strength of recommendations, 12% reported the searches for published studies, and all three criteria were met in only 5% of the guidelines. An assessment of 269 Chinese guidelines published from 1993 to 2010 found that only one guideline reported patients’ values and preferences, two reported external review, and 88% did not report sources of financial support for the guideline [7].

In 2017, the RIGHT (Reporting Items for Practice Guidelines in HealThcare) Working Group published the RIGHT reporting checklist for guideline reporting [8]. RIGHT has been widely implemented as the standard for guideline reporting criteria [9], encompassing seven main domains, namely basic information, background information, evidence, recommendations, review and quality assurance, funding declaration and management of interests, and other information. The 35-item checklist is a useful tool for guideline developers in clinical medicine and in public health as well for journal editors, peer reviewers and end users of guidelines.

WHO develops guidelines on a wide range of clinical and public health topics for use by various stakeholders worldwide. While the quality of WHO guidelines has been studied [10–13], to date, no study has examined the quality of reporting of WHO guidelines. Thus, the objective of this study was to examine the completeness and quality of reporting in WHO guidelines using the RIGHT reporting checklist.

## Methods

### Eligibility criteria and study selection

We obtained a list of all documents approved by the WHO Guidelines Review Committee (GRC), a quality oversight body for WHO guidelines. We included only guidelines that were submitted to the GRC in English. This list encompassed documents approved by the GRC from its inception in 2007 through 31 December 2017. Because not all documents in the list provided by WHO appeared to be guidelines, we developed staged eligibility criteria. First, we included only documents that contained specific recommendations for clinical practice or public health policy. Second, we included only documents with original recommendations, i.e. those not referencing another (source) guideline. If the document had such a reference, it was considered a derivative product and was excluded from our analysis as derivative products are not reasonably expected to report the same level of detail as the original guideline. Third, we excluded several documents that contained specific recommendation(s) but appeared to be derivative products although they did not reference a source guideline.

When a guideline was updated, we included the newest edition and examined prior versions for additional information. We downloaded all documents pertaining to each guideline from the WHO website (http://www.who.int/en/).

### Data extraction and analysis

Before starting data collection, we conducted three rounds of training on the RIGHT reporting checklist and four pilot tests of assessment among all the appraisers until they understood and agreed on the assessment items. Data were then formally extracted into a predesigned form by three pairs (QZ and YT, NY and CQ, BW and YX) of independent, trained researchers and any disagreements were resolved through discussion with a third researcher (XW). One author (XW) also verified all data extraction and any discrepancies were further discussed with the original extractors. The following data were extracted: (1) basic information about the guideline, including topic, developers and publication year; and (2) all content corresponding to items in the RIGHT reporting checklist [8].

We rated each of the 34 (out of 35) RIGHT items with dichotomous options — ‘reported’ or ‘not reported’. The ‘reported’ option was used when the relevant information was provided in the guideline, whereas ‘not reported’” indicated that the relevant information could not be found or was unclear. RIGHT item #13a (‘Provide clear, precise and actionable recommendations’) is not applicable at the level of the guideline because it must be assessed for each individual recommendation; we therefore indicated ‘not applicable’ for all guidelines for this item and removed this item from both numerator and denominator when calculating overall proportions.

Summary statistics (frequencies and percentages) are provided for each guideline characteristic and for each RIGHT item. To analyse reporting trends, we explored the mean number of reported items per guideline by year. We identified ten items related to four domains (evidence, recommendations, funding and conflict of interest (COI)) as key items because of their presumed importance in assessing the quality or trustworthiness of guidelines (hereafter referred to as ‘key items’). We then analysed trends in the reporting of these key items and compared them between guidelines developed by WHO alone versus by WHO in partnership with other organisations and between guidelines that reported using GRADE to assess the certainty of the evidence and/or formulate the recommendation versus those that did not. Because we included all WHO GRC-approved guidelines from 2008 to 2017, we report descriptive statistics only; our goal was not to infer or predict the characteristics of other cohorts of guidelines. We extracted and analysed the data using Excel 2016.

## Results

We obtained a list of 239 records approved by the GRC from its inception until 31 December 2017. A total of 182 guidelines fulfilled eligibility criteria, while 57 were excluded because they were not guidelines according to our criteria (*n* = 30), had been updated (*n* = 18), or were as yet unpublished or were published in 2018 (*n* = 9). Table 1 summarises the characteristics of the included guidelines.

**Table 1:** Characteristics of WHO guidelines (*n* = 182)

Characteristics		Number (%)
Publication year	2008	10 (5)
	2009	21 (12)
	2010	14 (8)
	2011	24 (13)
	2012	19 (10)
	2013	13 (7)
	2014	15 (8)
	2015	19 (10)
	2016	29 (16)
	2017	18 (10)
Developer	WHO	164 (90)
	WHO in partnership with other organisations	18 (10)
Reported using GRADE to assess and/or formulate the recommendation	Yes	148
	No	34
Topic	Infectious diseases	86 (47)
	Maternal and child health	34 (19)
	Nutrition, exercise and chronic disease prevention	21 (12)
	Public health emergencies including pandemics	6 (3)
	Environment and health	6 (3)
	Smoking and substance abuse	6 (3)
	Cancer	5 (3)
	Mental health and neurologic disorder	4 (2)
	Health policy	3 (2)
	Non-communicable diseases	3 (2)
	Disability and frailty	2 (1)
	Others	6 (3)
Guideline end-user^a^	Programme managers/staff	123 (68)
	Policy-makers	121 (66)
	Healthcare workers	114 (63)
	Technical/financial supporters	45 (25)
	National advisory board/government sectors	48 (26)
	Patient/consumer/public group or community	27 (15)
	Non-governmental organisation	23 (13)
	Researcher/academic staff	10 (5)
	Industry	9 (5)
	Training providers or facilitators	8 (4)
	Other	20 (11)
	Not reported	9 (5)

^a^One guideline may have several types of end-users

### Overall reporting of RIGHT reporting checklist items

**Fig. 1:**
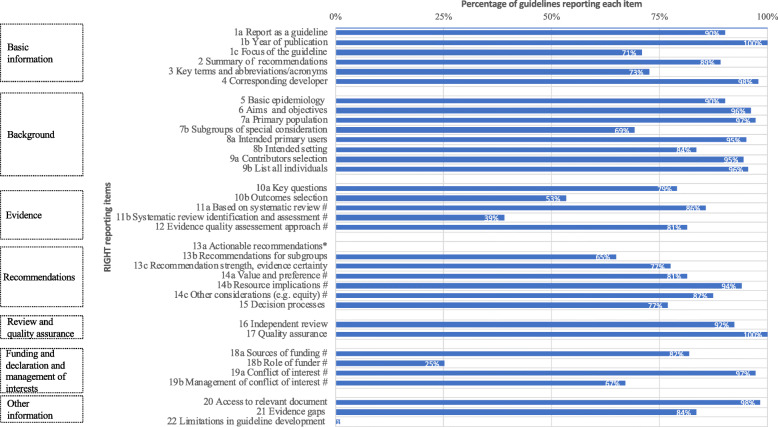
Reporting of RIGHT items in WHO guidelines. ^#^ Key items; * Not applicable for a guideline as a whole. *COI* conflict of interest

A total of 25 out of 34 RIGHT items were reported in 75% or more of WHO guidelines, 6 items were reported in 50–75%, and 3 (‘methods used to identify the systematic review’, ‘the role of funders’ and ‘limitation of guideline’) were mentioned in only 39%, 25% and 1%, respectively (Fig. 1). On average, WHO guidelines reported 25 of 34 (74%) items (median 27, range 10–31 items).

### Reporting according to each domain of the RIGHT reporting checklist

#### Basic information

Overall, more than 50% of WHO guidelines reported all the items in this domain and 164 (90%) reported the document type in the title. However, the descriptor for the document type varied considerably, including guideline(s) (*n* = 105), recommendation(s) (*n* = 27), guidance (*n* = 15), policy statements (*n* = 5), and a variety of other terms (manual, rapid advice, handbook, statement, guide, toolkit, technical paper). All the guidelines reported the publication year, either in the title or subtitle, elsewhere on the cover page, or on the copyright page. A glossary or a list of acronyms or abbreviations was provided in 132 (73%) guidelines, while 63 (35%) reported both a glossary and a list.

#### Background

All the items in this background domain were reported in more than 70% of guidelines; 175 (96%) guidelines clearly reported the aim(s) or specific objectives of the guideline. Some guidelines combined the aims and objectives while others distinguished them [14, 15]; 172 guidelines (95%) clarified the roles and responsibilities of contributors, while only 32 (18%) mentioned the process for selecting contributors.

#### Evidence

A total of 156 (86%) guidelines stated that the recommendations were based on systematic reviews, including 39 based on existing systematic reviews and 39 on new systematic reviews, while 78 used both. However, only 71 (39%) indicated the methods used to identify existing systematic reviews. All 148 (81%) guidelines that described the approach for assessing the certainty of evidence used the Grading of Recommendations, Assessment, Development and Evaluation (GRADE) approach.

#### Recommendations

Overall, 121 (66%) guidelines reported both the strength of recommendations and the certainty of evidence, whereas 18 (10%) reported only the strength of recommendations and 2 (<1%) reported only the certainty of evidence. Regarding the rationale for recommendations, many guidelines reported considering various important factors in addition to benefits and harms of the intervention without mentioning the methods used to collect this information. For example, 148 guidelines (81%) reported considering values and preferences while only 32 (18%) reported the methods used to collect this information. Similarly, 171 (94%) reported that cost and resource implications contributed to the recommendation but only 68 (37%) noted the sources for these data.

#### Review and quality assurance

A total of 168 (92%) guidelines reported information about the external review of the draft final guideline; 116 (64%) guidelines reported the peer reviewers, 123 (68%) described the review process and 56 (31%) mentioned management of the feedback, while only 33 (18%) reported all 3 of these components. We did not collect specific information from each guideline on quality assurance because we only included GRC-approved WHO guidelines. All such guidelines have undergone an independent quality assurance process, which is indicated on the website where these guidelines were sourced [16].

#### Funding and declaration and management of interests

The funding sources was reported in 149 (82%) guidelines, including 46 (25%) that stated the role of funders in specific stages of development and 2 that mentioned funding for the dissemination and implementation of recommendations. Declarations of interest for all contributors were reportedly obtained in 177 (97%) guidelines, of which 37 declared no COI. Of the 140 guidelines with a COI noted among contributors, 122 (87%) reported the management of COIs.

#### Other information

The sites where relevant documents could be accessed and gaps in the evidence were respectively reported in 98% and 84% of guidelines. On the other hand, only 2 (<1%) guidelines reported limitations, for example, *“A limitation of the search method was its restriction to guidelines in English. Also, there was no grey literature search*” [17].

#### Reporting by year of publication, developers and use of GRADE

**Fig. 2:**
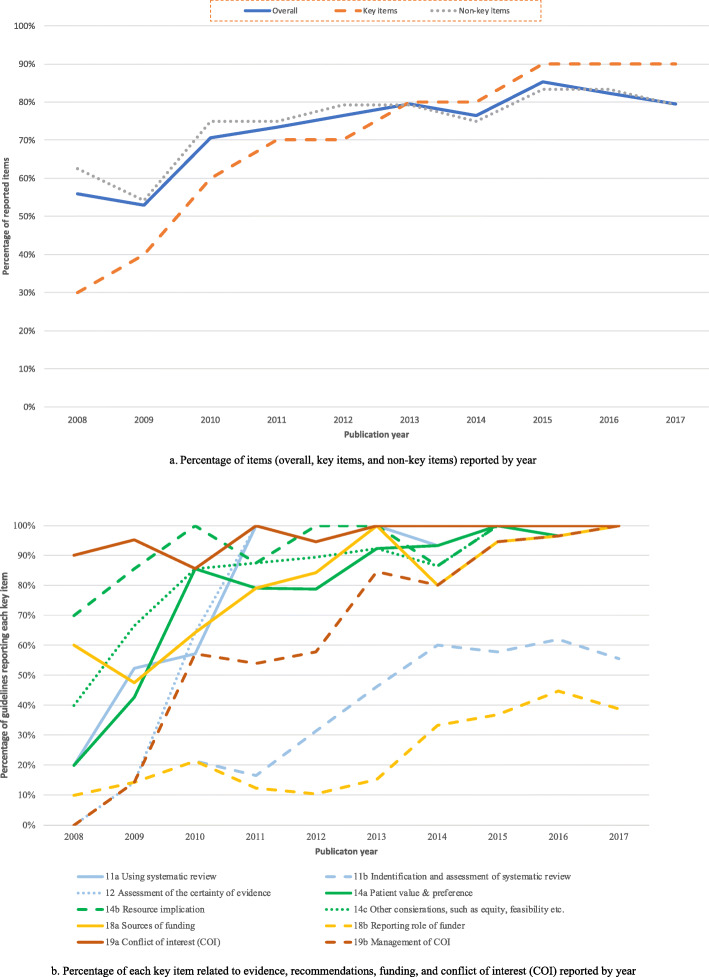
(**a**) Percentage of items (overall, key items and non-key items) reported by year. (**b**) Percentage of each key item related to evidence, recommendations, funding and conflicts of interest (COI) reported by year

We observed an increase over time in the percentage of items reported with a more remarkable increase in key items than in non-key items (Fig. 2a). The trends for each key item all indicated an increase in reporting over time (Fig. 2b).

**Fig. 3:**
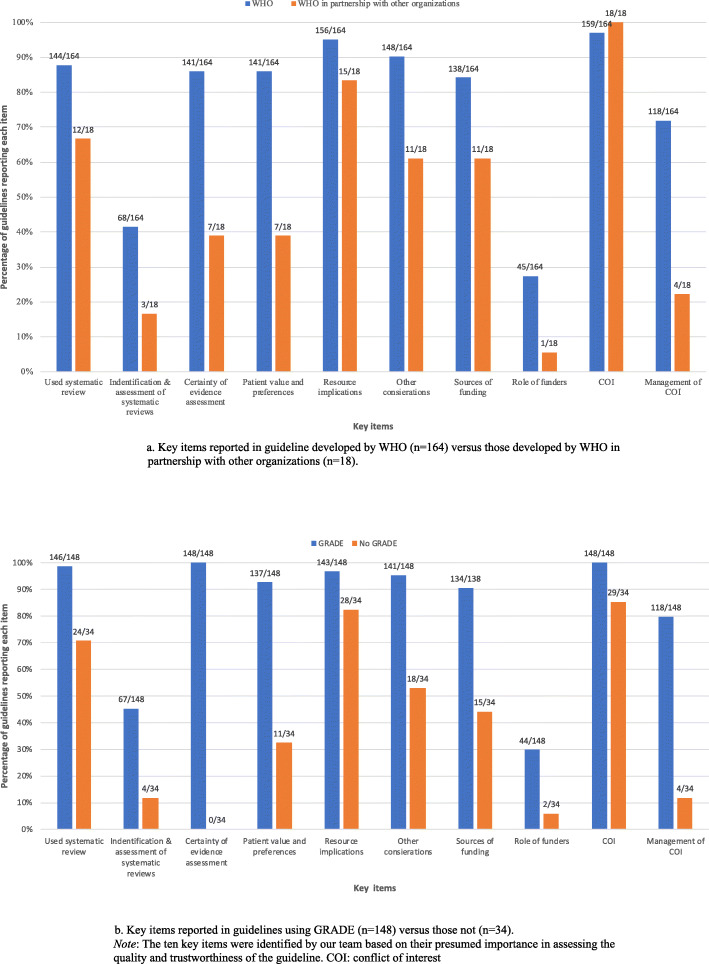
(**a**) Key items reported in guidelines developed by WHO alone (*n* = 164) versus those developed by WHO in partnership with other organisations (*n* = 18). (**b**) Key items reported in guidelines using GRADE (*n* = 148) versus those not (*n* = 34). (Note: The ten key items were identified by our team based on their presumed importance in assessing the quality and trustworthiness of the guideline). *COI* conflict of interest

We compared the reporting of key items between guidelines developed by WHO alone and by WHO in partnership with other organisations and found that reporting of all key items slightly favoured those developed by WHO alone, except for reporting of COIs, where 100% of partnered guidelines reported COIs (Fig. 3a). Among the guidelines that reported using GRADE versus those that did not, we observed better reporting of all key items in guidelines using GRADE (Fig. 3b).

## Discussion

This is the first comprehensive assessment of the reporting of WHO guidelines. We examined 182 WHO guidelines using the RIGHT reporting checklist. On average, 25 RIGHT items were reported in each guideline and 75% of guidelines reported more than 70% (25 out of 34) of items. The items in the basic information, background, recommendations, and review and quality assurance were reported in a high percentage of guidelines. On the other hand, the process of evidence preparation, the role of funders and the limitations of the guideline were reported in less than 50% of guidelines. We noted an increase in overall reporting completeness over time.

WHO guidelines compare favourably to prior analyses of guidelines developed by other entities — WHO adhered to a median of 79% of RIGHT items, while cohorts of Croatian and European practice guidelines both adhered to fewer than 50% of RIGHT items [18]. A study using the Checklist for the Reporting of Updated Guidelines in 2017 found that the methods used for assessing the quality of evidence and external review were reported in 77% and 38% of the updated guidelines from North America, Europe and Asia, respectively [9], compared with 81% and 68% in WHO guidelines.

Although most items were reported in many WHO guidelines, there is significant room for improvement. First, labels for guidelines varied, with more than 10 different terms used to represent a ‘guideline’. This could confuse users as to the nature of the document and could make it difficult for end users to retrieve and for indexers to categorise these documents [19]; this in turn may contribute to suboptimal dissemination and health impact [20, 21].

Second, items included in the RIGHT domain of evidence were reported at relatively low rates compared with other domains. Several studies using the Appraisal of Guidelines for Research and Evaluation (AGREE) II tool [22–25] to evaluate the methodological quality of guidelines produced by other organisations also report that evidence identification and evaluation are often problematic, which could mirror the reporting state and vice versa. In addition, a few WHO guidelines reported using the GRADE approach in their methods yet did not report the certainty of evidence or the strength of recommendations. Similarly, guideline groups reported considering values and preferences of target populations and resources implications when formulating recommendations, yet the guideline did not report how evidence related to these considerations was gathered and assessed. Because the assessment of guideline methodological quality relies on documented information [25, 26], developers need to pay more attention to the reporting of the methods used.

Third, while information about funders and declarations of interest was provided in more than 80% of the guidelines, the role of funders and the management of COIs were insufficiently reported. Similar findings have been noted previously in WHO guidelines [10]. Furthermore, only two guidelines reported the funding sources for dissemination and implementation plans. One possible explanation is that guideline developers are often not directly involved in the uptake and implementation of their recommendations, which is often the responsibility of national- or subnational-level programme managers who develop implementation plans and tools for their local context [27].

The reasons for the improvements noted in the reporting of items related to evidence, recommendation, funders and COI management over the last 10 years are unclear; however, the increased focus on adequate reporting in the health sciences may be having an impact. Improvements over the last decade in the quality of processes and methods used in WHO guidelines have also been reported [10–12] and may reflect WHO’s continuous efforts to advance their methods and procedures and the rigorous quality assurance process overseen by the GRC. The use of GRADE may also improve reporting, given its structured approach to assessment and presentation of the body of evidence and formulation of recommendations [28]. However, almost all of the 34 guidelines that did not use GRADE were published before 2010 and this comparison might be confounded by year of publication given that reporting quality improved over time.

The organisations involved in guideline development may influence reporting [25] and we found that the reporting on evidence, recommendation, funders and management of COIs was better in guidelines developed by WHO alone than in those co-developed with other organisations, although both the sample size and the differences detected were small. The potential reasons for this need to be explored but might include difficulties aligning the requirements of other organisations with WHO’s requirements.

Our findings have implications for guideline developers in other organisations, suggesting common areas where improvements are needed, regardless of the nature of the guideline producer. In addition, end users of WHO and other guidelines need to be alert to suboptimal reporting when they assess, select and implement guidelines. Just as reporting has improved for clinical trials following the publication and widespread uptake of CONSORT [29], we hope that the increased use of RIGHT for guidelines will have a similar impact over time.

### Considerations when using the RIGHT reporting checklist

WHO guidelines have rather unique processes, requirements and specifications [30] such as mandatory executive summaries, uniform front-matter and institutional authorship with acknowledgement of individual contributors. We used RIGHT items as the reference; however, we had to adapt some items. For example, we extracted the email addresses for general correspondence rather than listing ‘corresponding authors’. We also judged the quality assurance process according to WHO’s stated overall process and methods [30] as this information was not generally found in each specific guideline. Similarly, users of RIGHT to evaluate existing guidelines need to make appropriate modifications at the outset of any such processes.

Some information could only be found in an older version of a WHO guideline. For instance, the main recommendations and list of abbreviations and acronyms in a 2015 HIV guideline [31] could only be found in the 2011 version [32]. The same issue occurs in guidelines from other organisations, such as the anti-thrombotic guidelines from the American College of Chest Physicians, where the latest (10th) version [33] refers back to the 9th [34] for some information. We thus had to search previous editions of some WHO guidelines for relevant information, including evidence summaries, recommendations that were unchanged and still current, and guideline development groups and their COIs [35]. This approach is not optimal for transparency and end users may be unclear as to which recommendations and evidence are current and they may have to spend additional time finding the information they need.

Some sub-items in the RIGHT reporting checklist comprise multiple components. For example, item 11b concerns reporting the use of existing systematic reviews in guidelines, including search strategies, selection criteria, risk of bias and updating information. We found that many guidelines reported some but not all of the listed criteria and thus it was difficult to provide a single assessment of this sub-item. This issue has been noted for other reporting tools such as the Preferred Reporting Items for Systematic reviews and Meta-Analyses (PRISMA) statement [36, 37]. As noted by Yao et al. [38], RIGHT and the AGREE Reporting Checklist both apply to practice guidelines and, although many of the items in these two tools overlap, there are differences, for example, 12 items are listed in RIGHT only, while 4 are unique to AGREE, including evidence selection criteria, updating procedure, facilitators and barriers to application, and monitoring/auditing criteria. Given the importance of the evidence review process in the development of a trustworthy guideline, the RIGHT working group is planning to extend the checklist for reporting systematic reviews in practice guidelines to provide a more detailed examination of each key aspect of reporting [39].

### Limitations

There are several limitations to our study. First, we included a limited cohort of WHO guidelines, including only guidelines approved by the GRC, where the first version was published in English. WHO produces some guidance documents that are not submitted to the GRC and produces guidelines in other languages (in addition to the translation of many English-language guidelines into other languages). Second, we had to adapt RIGHT for item 13a regarding clear, precise, actionable recommendations because we could not make an overall judgement for each guideline as each individual recommendation should be evaluated on its own merits [40, 41] and our objective was to assess the reporting quality of each guideline as a whole. The RIGHT working group is planning an extension for reporting recommendations to address this issue [39]. Finally, we examined the proportion of all RIGHT items reported in each year to explore trends over time; however, we deem some reporting items to be potentially more important than others, thus the comparison of proportions, which assumes each item is of equal importance for assessing trustworthiness, should be interpreted with caution.

### Next steps

This is the first paper to investigate the reporting quality of WHO guidelines and our team is now working on a follow-up study, which will include the assessment of WHO guidelines published after 2017 with further analysis comparing the quality of WHO guidelines published before and after RIGHT was published.

## Conclusions

The majority of WHO guidelines included most RIGHT reporting items and reporting quality has improved over time. Further improvements are needed in the reporting of limitations in guideline development, the role of funders, and the identification and assessment of the evidence. These findings are likely relevant to guidelines produced by other organisations, although some RIGHT items may need to be modified when applied to guidelines produced by organisations with unique formats such as WHO.

## Data Availability

The datasets used and analysed in this study are available from the corresponding author on reasonable request.

## Abbreviations

GRC: Guidelines Review Committee
RIGHT: Reporting Items for Practice Guidelines in Healthcare
GRADE: Grading of Recommendations, Assessment, Development and Evaluation
COI: Conflict of interest
AGREE: Appraisal of Guidelines for Research and Evaluation
